# Modeling of Linear Die Filling Based on Dimensional Analysis Using DEM-CFD Methods

**DOI:** 10.3390/ma18143261

**Published:** 2025-07-10

**Authors:** Jie Li, Sunsheng Zhou, Shiyan Yan, Yuanqiang Tan, Jiangtao Zhang

**Affiliations:** 1 Institute of Manufacturing Engineering, Huaqiao University, Xiamen 361021, China; 21011080007@stu.hqu.edu.cn (J.L.); 23011080020@stu.hqu.edu.cn (S.Z.); 22014080103@stu.hqu.edu.cn (S.Y.); 2 National & Local Joint Engineering Research Center for Intelligent Manufacturing Technology of Brittle Material Products, Huaqiao University, Xiamen 361021, China; 3 Henan Key Laboratory of Superhard Abrasives and Grinding Equipment, Henan University of Technology, Zhengzhou 450001, China; zhangjiangtao@haut.edu.cn

**Keywords:** powder die filling, dimensionless processing, DEM-CFD, critical value, semi-empirical modeling

## Abstract

Linear die filling is currently widely employed in industries. However, there is no comprehensive and systematic model to describe the powder die filling process. This paper utilizes dimensional analysis to extract and analyze various factors that affect the flow characteristics of powder based on DEM-CFD simulations. Several dimensionless parameters including the ratio of particle size to die depth ($d_{p}h_{D}^{-1}$), solid density number ($\rho_{p}\rho_{g}^{-1}$), shoe speed number ($v\rho_{g}L_{D}\mu^{-1}$), and force number ($G_{p}F_{Drag}^{-1}$) were proposed based on the Pi theorem. The results showed that the filling ratio δ increased with the increase in $d_{p}h_{D}^{-1}$ and $\rho_{p}\rho_{g}^{-1}$ due to $G_{p}F_{Drag}^{-1}$ rising. But it decreased with the increase in $v\rho_{g}L_{D}\mu^{-1}$ due to the shortening of effective filling time. Finally, a semi-empirical modeling of linear die filling was developed, taking the critical value ${\left(d_{p}h_{D}^{-1}\right)}_{90}$ as the dependent variable and the solid density number ($\rho_{p}\rho_{g}^{-1}$) and shoe speed number ($v\rho_{g}L_{D}\mu^{-1}$) as independent variables. Hence, this model provides a new approach to computing the smallest shoe speed and designing the sizes of dies based on measurable material properties under complete die filling.

## 1. Introduction

Die filling serves as the fundamental procedure determining the quality and performance of powder products as it directly controls powder fill mass homogeneity while concurrently affecting the green compact’s structural integrity [1]. There are some factors influencing quality and strength, such as the filling process [2], powder properties [3], geometrical parameters [4,5,6], and the airflow [7,8,9,10] inside the die. Generally, powder properties determine flowability, which influences filling efficiency significantly; geometrics, mainly referring to the shape and size of dies, also play a role; and airflow hinders the falling of particles. The selection of process parameters is mainly based on experience, which often leads to suboptimal parameter combinations. In the worst case, incomplete filling may occur, resulting in the quality of products not meeting usage requirements [11]. Up to now, there has been no comprehensive and systematic analysis or filling model to describe and instruct the filling process. Therefore, a comprehensive investigation and systematic research must be undertaken.

Linear die filling is a typical case. Researchers have analyzed operational protocols in device systems. Shoe speed is a major process parameter. The critical velocity proposed experimentally [3] is the maximum shoe speed at which complete filling just occurs, which is a classical model [6]. Furthermore, enhancing the flowability by improving powder properties is also a better choice. Large [12], near-spherical [3,11], and heavier particles [5] have better flowability. The flow features are different and generally divided into three types: nose flow, bulk flow, and intermittent flow [6]. Nose flow is observed as a phenomenon where powder migrates rearward along the shoe, forming nose flow, driven by inertial forces during shoe acceleration and powder-base frictional resistance. Nose flow allows surface particles to exhibit enhanced mobility while facilitating rapid air venting from the die, jointly boosting fill efficiency. As the nose flow front advances, shear-induced detachment occurs in powder layers that slide across the base plane, which is referred to as “bulk flow”. Furthermore, the bulk flow is dominant under high shoe speeds. Intermittent flows often occur when powders are cohesive [13], when the flowability is bad. The shape and depth of dies also have some influence on the filling ratio. For example, when powder is filled into a stepped mold, the corner gaps cannot be filled [14]. Schneider [13] found that the amount of powder filling the die in vacuum was more than that in the presence of air at the same shoe speed, and the vacuum conditions increased the critical velocity compared to air-filled systems. The differences between linear and rotary die filling were investigated by Zhong [15], and the results demonstrated that poor-flowing powders exhibit marginally superior fill performance in linear dies versus rotary configurations, whereas free-flowing powders achieve comparable results in both systems. In addition, the effects of moisture content [16] and agitator structure [17] on fill performance were studied.

In order to strengthen flowability, suction is introduced. Suction filling can improve filling efficiency under the same shoe speed condition compared to gravity filling, and the critical velocity in suction filling is generally less than that in gravity filling [4,18]. In addition, the improvement effect of suction filling is more pronounced for powders with poor flowability. For example, the flowability of cohesive powders is enhanced more significantly than that of free-flowing powders [19]. In addition, the tendency for segregation during suction filling is also reduced [20]. However, the cost of suction filling equipment is higher in industrialization. The majority of the above achievements are based on experimental research. Existing filling models derived from laboratory studies show limited transferability to industrial-scale engineering design. Therefore, the discrete element method (DEM) has been widely employed in handling bulk materials. Due to the non-negligible effect of air, computational fluid dynamics (CFD) coupled with the DEM is currently widely applied. The DEM-CFD method is employed extensively in multiple disciplines such as crop residue cleaning [21], materials conveying [22], throttle valve erosion [23], and particle deposition [24]. The reliability of this method has been extensively demonstrated.

The DEM-CFD method for linear die filling was investigated [5,8,9,25], and the analysis revealed that air had obvious impacts on powder flowability. Specifically, low-density and fine particles are more sensitive. Therefore, the air enhances the density segregation tendency and weakens the size segregation tendency. Furthermore, the particle’s sensitivity to the air could be described by a dimensionless parameter ξ, where particles can hence be classified into two regimes with a critical value of the dimensionless parameter ($\xi =9.56\times 10^{6}$). These include air-sensitive particles ($\xi <\xi_{C}$), for which air has a significant impact on powder flow, and air-inert particles ($\xi >\xi_{C}$), for which the impact of air can be neglected. However, the dimensionless parameter ξ cannot take the geometric parameters of the die into consideration, and for air-sensitive particles, there is no further study exploring the correlation between the dimensionless parameter ξ and the filling ratio.

Industrial particle systems exceed single-node computational feasibility, requiring distributed computing [26]. Consequently, the coarse-graining methodology (CGM) is implemented to achieve computationally tractable gas–solid flow simulations. The CGM simulations have been validated for dense granular regimes, such as bubbling fluidized beds [27,28], pneumatic conveying systems [29], and spouted beds [30,31]. In addition, the CGM has undergone rigorous verification and validation against benchmark cases [26,32], where the simulation results exhibit quantitative agreement with experimental measurements within a relative error of 3%. In addition, linear die filling based on the DEM-CFD method with CGM was introduced and verified by Xie [31].

Above all, these studies mainly focused on a single factor or aspect. The model of mass flow rate of linear die filling was established by Schneider [13] based on dimensional analysis, but the solid density was neglected. The critical velocity is only an indicator that cannot be directly used in industry due to its recalibration when the material changes. Schomberg et al. experimentally derived a fill process model for rotary tablet presses [11], which, however, is not applicable to linear filling.

Therefore, the applicability and promotability of the current models and findings on linear die filling are limited and cannot take all impact factors into account. In this paper, dimensional analysis was used to explore the complicated interactions among powder properties, process parameters, and geometric parameters in the presence of air. The coarse-graining DEM-CFD coupled method was adopted, and air-sensitive particles such as fine and lighter particles were focused on. The aim of the study is to reveal the mechanism of linear die filling and establish the correlations among these parameters comprehensively. Furthermore, developing a semi-empirical model to predict the filling results is the ultimate goal.

## 2. Numerical Modeling

This section presents details about the mathematical approach, including the discrete element method and computational fluid dynamics.

### 2.1. Discrete Element Method

The DEM is employed to model the motion of solid particles. In DEM simulations, contact forces between particles are introduced and Newtonian motion equations are solved to obtain physical information about the particles’ behavior over time. Forces and torques acting on each particle facilitate their translation and rotation, with the relevant equations shown below.

(1) $$m_{i}\frac{du_{i}}{dt}=\sum F_{C}+F_{D}+mg$$

and

(2) $$I_{i}\frac{dw_{i}}{dt}=\sum T_{i}$$

where $m_{i}$, $\mu_{i}$, $I_{i}$, and $w_{i}$ were the mass, linear velocity, moment of inertia, and angular velocity of particle *i*, respectively. ***g*** is the gravitational acceleration, $F_{C}$ is the contact forces between particles and wall, $F_{D}$ is the interaction force between particle and air (mainly drag force), and $T_{i}$ represents the torque arising from the tangential force $F_{t,i}$. $F_{n,i}$ and $F_{t,i}$ are the normal force and tangential force, respectively. Boldface symbols denote vector quantities.

In this study, the Hertz contact model is employed to determine the normal contact force on particles during contact, and the Mindlin and Deresiewicz model is used for the tangential force [33,34,35]. The normal component of the contact force is given by the following equation:

(3) $$F_{n,i}=-K_{n}\delta_{n}^{\frac{3}{2}}-C_{n}{\dot{\delta }}_{n}\delta_{n}^{\frac{1}{4}}$$

where $K_{n}$, $\delta_{n}$, and $C_{n}$ are the normal stiffness, normal overlap, and normal damping coefficient, respectively. The first term on the right-hand side of the equation represents the repulsive force on the particles, while the second term represents the energy dissipation before and after contact.

And the tangential force is defined as follows:

(4) $$F_{t,i}=\left\{\begin{array}{l} -K_{t}\delta_{t}-C_{t}{\dot{\delta }}_{t}\;\;\;\left(\left|F_{t,i}\right|<\mu \left|F_{n,i}\right|\right)\\ -\mu \left|F_{n,i}\right|\cdot u_{t}/\left|u_{t}\right|\;\;\left(\left|F_{t,i}\right|\geq \mu \left|F_{n,i}\right|\right) \end{array}\right.$$

where $K_{t}$, $\delta_{t}$, and $C_{t}$ represent the tangential stiffness, tangential overlap, and tangential damping coefficient.

The drag force $F_{D}$ between particles and air is given by

(5) $$F_{D}=\frac{1}{2}C_{D}\rho_{f}A_{i}\left|u_{f}-u_{i}\right|\left(u_{f}-u_{i}\right)$$

where $C_{D}$, $\rho_{f}$, $A_{i}$, and $u_{f}$ represent the drag coefficient, fluid density, projected area of particle *i* in the flow direction, and fluid velocity, respectively. *A_i_* is calculated as follows:

(6) $$A_{i}=\frac{\pi d_{i}^{2}}{4}$$

$d_{i}$ is the size of particle *i*. The Gidazpow, Bezburuah & Ding [36] drag correlation covers the entire range of solids (particle phase) volume fraction (from 0 up to the maximum packing limit) but presents a discontinuity at the point $\alpha_{f}=0.8$. To ensure a smoother transition between the Ergun [37] correlation in Equation (9) and the Wen & Yu [38] correlation in Equation (10), Huilin & Gidazpow [39] applied a blending function in Equations (7) and (8) to promote the connection based on the fluid volume fraction $\alpha_{f}$.

(7) $$C_{D}=\varphi C_{D,Ergun}+\left(1-\varphi \right)C_{D,Wen\&Yu}$$

where φ, $C_{D,Ergun}$, and $C_{D,Wen\&Yu}$ represent the blending parameter, the drag coefficient of Ergun, and the drag coefficient of Wen & Yu, which are written as follows:

(8) $$\varphi =\frac{1}{\pi }\arctan\left[150\times 1.75\left(0.8-\alpha_{f}\right)\right]+0.5$$

(9) $$C_{D,Ergun}=\frac{200\alpha_{p}}{\alpha_{f}\phi^{2}Re_{p}}+\frac{7}{3\phi }$$

(10) $$C_{D,Wen\&Yu}=\alpha_{f}^{-1.65}\max\left\{\frac{24}{\alpha_{f}Re_{p}}\left[1+0.15{\left(\alpha_{f}Re_{p}\right)}^{0.687}\right],0.44\right\}$$

where ϕ and ${Re}_{p}$ are the sphericity of the particle and the particle’s Reynolds number. In this research, a sphere particle is selected, ϕ=1. ${Re}_{p}$ is calculated as follows:

(11) $$Re_{p}=\frac{\rho_{f}\left|u_{i}-u_{f}\right|d_{i}}{\mu_{f}}$$

where $\mu_{f}$ is the dynamic fluid viscosity.

### 2.2. Computational Fluid Dynamics

The air is treated as a continuum and modeled using CFD, in which the continuity and momentum equations are considered.

(12) $$\frac{\partial \alpha_{f}}{\partial t}+\nabla \cdot \left(\alpha_{f}u_{f}\right)=0$$

and

(13) $$\frac{\partial }{\partial t}\left(\alpha_{f}\rho_{f}u_{f}\right)+\nabla \cdot \left(\alpha_{f}\rho_{f}u_{f}u_{f}\right)=-\alpha_{f}\nabla p-f+\nabla \cdot \left(\alpha_{f}\tau_{f}\right)+\alpha_{f}\tau_{f}g$$

where $\tau_{f}$ is the viscous stress tensor, **g** is the gravitational acceleration, and ***f*** is calculated by summing up the fluid–particle interaction force given in Equation (14) and divided by the volume of the CFD grid as

(14) $$f=\frac{\sum_{i=1}^{N}F_{D}}{V_{mesh-grid}}$$

where N is the number of particles in the CFD grid and *V_mesh-grid_* is the volume of the CFD grid. The two-way coupling between the solid and fluid phases can be implemented by Equation (12).

The geometric dimensions of the linear filling system are much larger than the actual particle sizes, resulting in an excessively large particle system. During the handling of bulk materials, the estimated number of particles can reach billions, making simulation computations too extensive. Therefore, this paper adopts a coarse-graining DEM for particle scaling. The coarse-graining DEM approach involves replacing many particles with the same properties (such as density and mass) with a single large particle, termed as coarse-grained particles after replacement [32]. In conjunction with fine particle sizes and equipment geometric dimensions, this study selects a scale-up factor of 10 for the coarse-grained particle model, which is explained in Section 3. Details regarding the scaling criteria of particle material properties can be found in these studies [26,29,31,40,41,42,43,44].

### 2.3. Simulation Conditions

**Figure 1:**
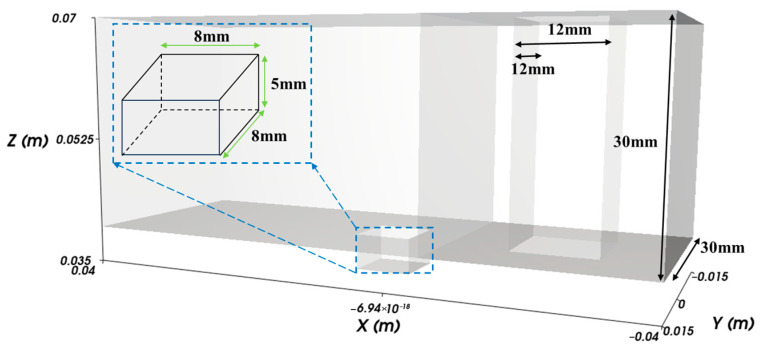
Linear die filling system.

The geometry of the powder die filling system is illustrated in Figure 1. The system consisted of a shoe, a table, and a die. The domain size of the system was 80 mm, 30 mm, 30 mm in the x, y, and z directions. The size of the shoe was 12 mm, 12 mm, and 30 mm in the x, y, and z directions. The die was a rectangular cavity, and the size of the die was 8 mm, 8 mm, and 5 mm in the x, y, and z directions.

**Figure 2:**
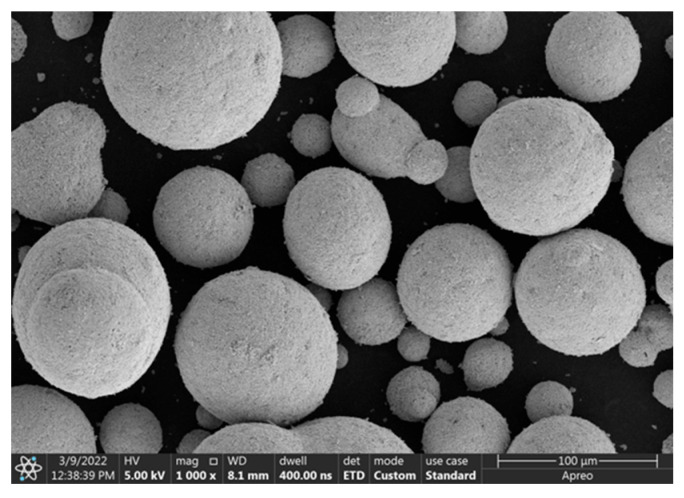
The SEM images of WC-10Co particles.

**Figure 3:**
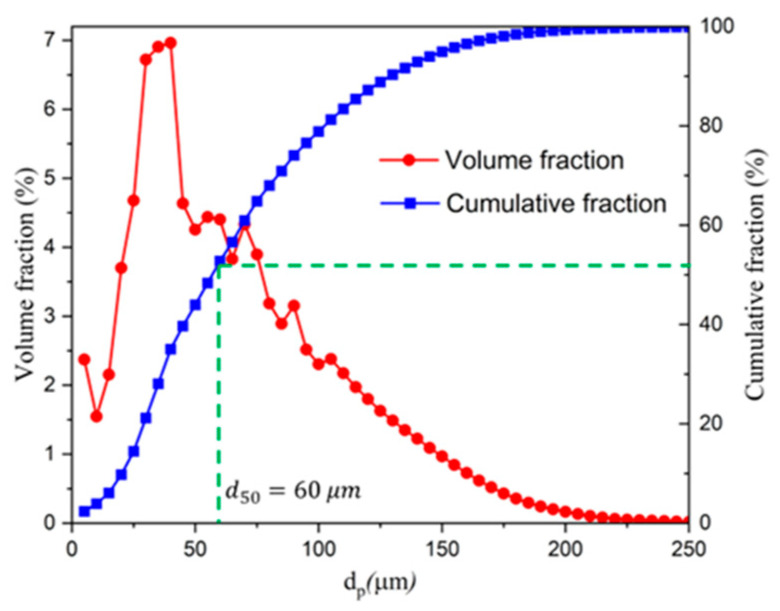
The size distribution of WC-10Co particles.

In DEM, parameter calibration is key to ensuring the accuracy and reliability of numerical results. In our research, WC-10Co, in which the powder material is composed of a WC matrix with a 10 wt% Co binder phase, was selected as the main subject. The solid density of the particles is 11,360 kg/m^3^, which is a relatively heavy particulate material. The density range of 3360~11,360 kg/m^3^ was selected in our research, which covers the density of all particulate materials. And the interval of the density range is 2000 kg/m^3^. Figure 2 shows the SEM images of the powder particles. And it can be seen that the particles have good sphericity. Therefore, sphere particles were used in DEM. Figure 3 shows the size distribution of particles from a dynamic particle size and shape analyzer, “CAMSIZER X2”. By combining it with the sensitivity coefficient of particles to air, proposed by Guo [8], it is calculated that when the particle size does not exceed 60 μm, the particles are all air-sensitive. Therefore, the size range of 20~60 μm was considered and used in DEM, with a size interval of 10 μm. The sensitivity coefficient was calculated as follows:

(15) $$\zeta =\frac{\rho_{a}\left(\rho_{s}-\rho_{a}\right)gd_{p}^{3}}{\eta^{2}}\cdot \frac{\rho_{s}}{\rho_{a}}$$

**Figure 4:**
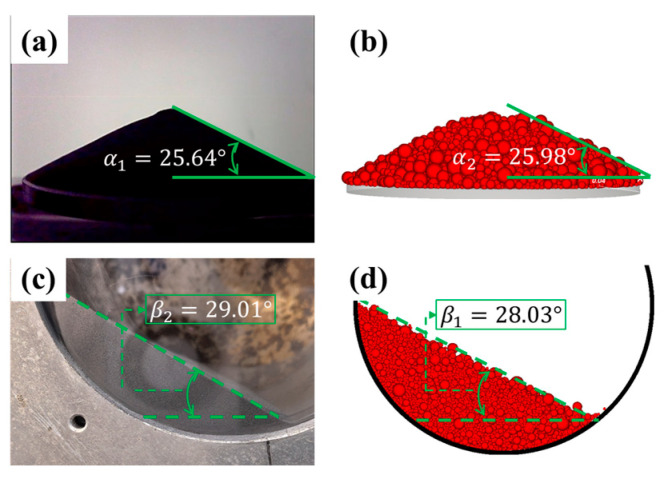
The static repose angle in experiment (**a**) and DEM simulation (**b**) and the dynamic repose angle in experiment (**c**) and DEM simulation (**d**).

The contact model of normal force was a Hertzian spring–dashpot model, and the contact model of tangential force was a Mindlin–Deresiewicz model [35]. The static and dynamic repose angles were used to calibrate and validate the inertial friction and rolling resistance, as shown in Figure 4.

During the calibration, the CGM (coarse-graining model) was adopted and the scale-up factor was set to 10. In addition, the gas density was set to 1.225 kg/m^3^, and the viscosity of the air was set to $1.75\times 10^{-5}$ Pa s in CFD. The other parameters in DEM, such as restitution coefficients and shoe speed, were determined from a dropping test and industrial experience. The parameters in DEM and CFD coupling are listed in Table 1.

In order to verify the accuracy of the scale-up factor and select a suitable one, in our research, the CGM scale-up factors were set to 1, 4, 6, 8, and 10, as shown in Table 1, where the particle size is 40 μm, the solid density is 7360 kg/m^3^, and the shoe speed is 0.150 m/s. The simulation of die filling was conducted with different scale-up factors.

**Figure 5:**
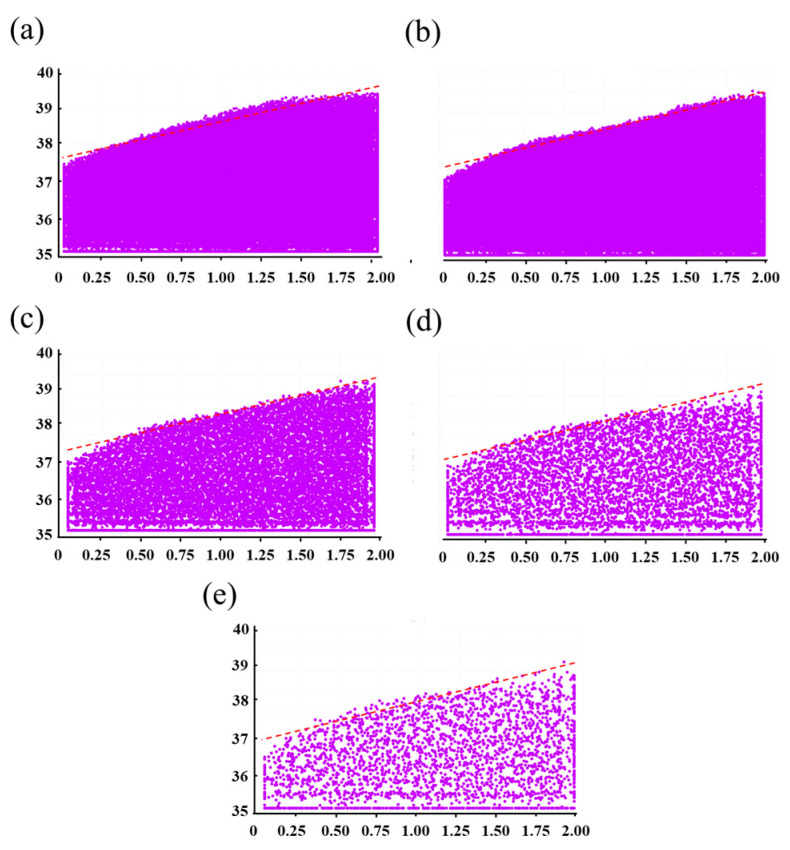
The scatter plot of powders settled inside the die with different scale-up factors: (**a**) k=1, (**b**) k=4, (**c**) k=6, (**d**) k=8, and (**e**) k=10, where the red line represents the fitted line for the profile slope.

Figure 5 presents a typical scatter plot of powders inside the die after filling. When different scale-up factors from Figure 5b–e were used, a similar phenomenon was observed: the pile surface of powders formed and sloped up from the left side to the right side, similarly to the initial particle system in Figure 5a. The angle of the pile surface was selected as an indicator and maintained in a narrower range from 14.52° to 14.77°. Table 2 showed the filling ratios of the CGM with different scale-up factors. Although the scale-up factors changed in the selected range from 1 to 10, the filling ratio always remained around 50.7%. Therefore, the coarse-grain system has similar characteristics to the original system, and a scale-up factor of 10 was selected to ensure an acceptable computational load of the simulation.

To analyze the influence of the breadth depth ratio of dies, the widths of the dies were 6 mm, 7 mm, and 8 mm, respectively. The depths of the dies were 5 mm, 6 mm, and 7 mm, respectively.

For the initial condition in each simulation, the particles were generated randomly inside the shoe cavity. In order to make a fair comparison among different conditions, the CFD grid size was set equivalently in all cases. As a matter of course, the CFD grid size should be slightly larger than the maximum particle size due to the volumetric diffusion technique. The time step for DEM was self-adaptive, varying from $2\times 10^{-7}$ to $1\times 10^{-6}$ s, and for gas dynamics, it was set to $1\times 10^{-5}$ s. ROCKY4.4-DEM was adopted to simulate the motion of particles, and FLUENT2020R1-CFD was used to calculate the air dynamics.

## 3. Results and Discussion

The key physical variables related to the induced airflow $\mu_{f}$ were obtained by Li [45,46], and the semi-empirical equation for induced airflow velocity were derived based on similitude theory. In addition, the model of linear die filling on mass flow rate was derived by Schneider based on dimensional analysis. Following this method, the parameters affecting die filling are presented as follows: shoe speed v, the depth of the die $h_{D}$, size of the cross section $L_{D}$, density of air $\rho_{g}$, air viscosity $\mu_{g}$, solid density $\rho_{p}$, particle size $d_{p}$, particle gravity $G_{p}$, drag force $F_{Drag}$, and filling ratio δ. All physical variables may be described using three basic dimensions: [L], [M], and [T]. $\rho_{g}$, $L_{D}$, and μ were selected as independent variables with dimensions of $\left[ML^{-3}\right]$, [L], and $\left[ML^{-1}T^{-1}\right]$. The dimensions of the remaining six variables v, $h_{D}$, $\rho_{p}$, $d_{p}$, $G_{p}$, and $F_{Drag}$ were $\left[LT^{-1}\right]$, [L], $\left[ML^{-3}\right]$, [L], $\left[MLT^{-2}\right]$, and $\left[MLT^{-2}\right]$. The theory of Buckingham Pi was used, and the shoe speed v can be written as follows: $v=\pi_{1}\left(\rho_{g}^{a}L_{D}^{b}\mu_{g}^{c}\right)$. The corresponding dimensional equation can be written as follows: $[LT^{-1}]={\left[ML^{-3}\right]}^{a}{\left[L\right]}^{b}{\left[ML^{-1}T^{-1}\right]}^{c}=M^{a+c}L^{-3a+b-c}T^{-c}$. It can be obtained by solving the equation: a=−1, b=−1, c=1, $\pi_{1}=v\rho_{g}L_{D}\mu_{g}^{-1}$. In the same manner, the π-terms of the remaining five physical variables can be obtained: $\pi_{2}=h_{D}L_{D}^{-1}$, $\pi_{3}=\rho_{p}\rho_{g}^{-1}$, $\pi_{4}=d_{p}L_{D}^{-1}$, $\pi_{5}=G_{p}\rho_{g}\mu^{-2}$, and $\pi_{6}=F_{Drag}\rho_{g}\mu^{-2}$. Since the fill ratio δ was inherently a dimensionless parameter, no transformation was required. In addition, by letting $\pi_{2}/\pi_{4}$, $\pi_{5}/\pi_{6}$, four dimensionless parameters were obtained: $d_{p}h_{D}^{-1}$, $\rho_{p}\rho_{g}^{-1}$, $v\rho_{g}L_{D}\mu_{g}^{-1}$, and $G_{p}F_{Drag}^{-1}$. The physical meanings of the four dimensionless parameters were as follows: the ratio of particle size to die depth $d_{p}h_{D}^{-1}$, the solid density number $\rho_{p}\rho_{g}^{-1}$, the shoe speed number $v\rho_{g}L_{D}\mu_{g}^{-1}$ and the force number $G_{p}F_{Drag}^{-1}$.

The ratio $d_{p}h_{D}^{-1}$ combined particle size $d_{p}$ and die depth $h_{D}$. Generally, the larger the value of $d_{p}h_{D}^{-1}$ is, the less sensitive the particle is to air. To some extent, the airflow inside the die cavity will hinder the filling of particles. In addition, the solid density number $\rho_{p}\rho_{g}^{-1}$ means that particles heavier than air are more insensitive to airflow. And the shoe speed number $v\rho_{g}L_{D}\mu_{g}^{-1}$ consisting of the process parameter (shoe speed v), air properties, and die size (die width) denotes that a higher shoe speed needs to be matched with a larger die width to achieve complete filling. Above all, the filling ratio δ will be a function that is related to the ratio number $d_{p}h_{D}^{-1}$, solid density number $\rho_{p}\rho_{g}^{-1}$, and shoe speed number $v\rho_{g}L_{D}\mu_{g}^{-1}$, which is as follows:

(16) $$\delta =f\left(d_{p}h_{D}^{-1},\rho_{p}\rho_{g}^{-1},v\rho_{g}L_{D}\mu_{g}^{-1}\right)$$

Taking the filling ratio δ as the evaluation index, the influence mechanism and law of each dimensionless parameter on the die filling process were analyzed. The fill ratio δ was calculated as follows:

(17) $$\delta =\frac{m_{r}}{m_{c}}=\frac{V_{r}\cdot \rho_{b}}{V_{D}\cdot \rho_{b}}=\frac{V_{r}}{V_{D}}$$

where $m_{r}$, $m_{c}$, $V_{r}$, $V_{D}$, and $\rho_{b}$ are actual filled mass, complete filled mass, actual filled volume, volume of die cavity, and bulk density of particles, respectively. The actual filled volume $V_{r}$ is directly related to particle size $d_{p}$, and the volume of die cavity $V_{D}$ is related to die depth $h_{D}$ and die width $L_{D}$. Therefore, the left of Equation (17) can be simplified as $\delta =G_{1}(d_{p}h_{D}^{-1})/G_{2}(L_{D})$. Combining with Equation (16), Equation (18) can be induced as follows:

(18) $$G_{1}(d_{p}h_{D}^{-1})=f^{\prime }\left(d_{p}h_{D}^{-1},\rho_{p}\rho_{g}^{-1},v\rho_{g}L_{D}\mu_{g}^{-1}\right)$$

The ratio number $d_{p}h_{D}^{-1}$ was extracted separately, and Equation (19) was further derived as follows:

(19) $$d_{p}h_{D}^{-1}=f\prime \prime \left(\rho_{p}\rho_{g}^{-1},v\rho_{g}L_{D}\mu_{g}^{-1}\right)$$

### 3.1. The Effect of Ratio Number

**Figure 6:**
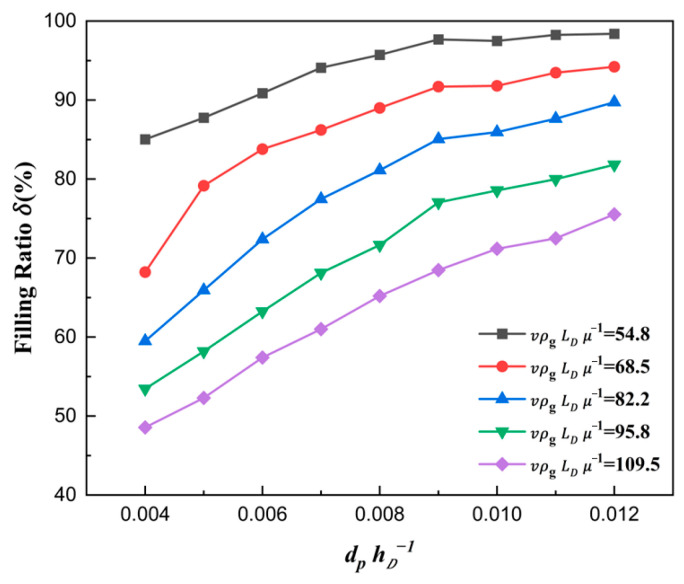
The filling ratio δ in different ratio numbers for $\rho_{p}\rho_{g}^{-1}=6008$.

**Figure 7:**
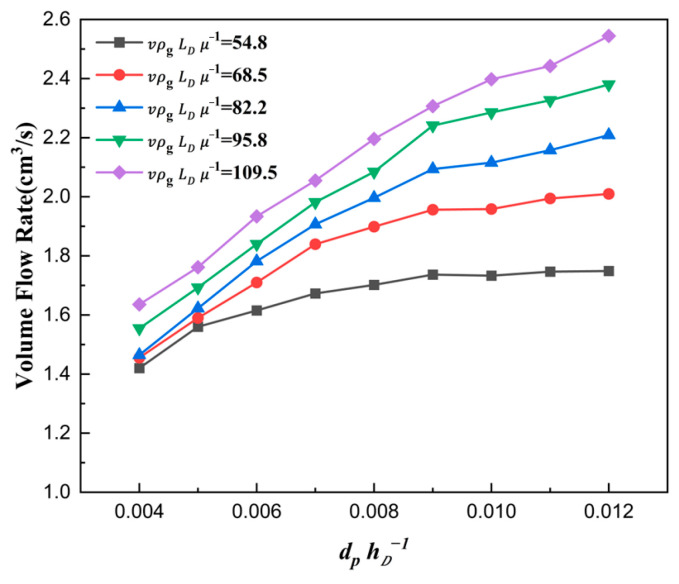
The volume flow rate of particles filled in different ratio numbers for $\rho_{p}\rho_{g}^{-1}=6008$.

The curve obtained by using Equation (17) is plotted in Figure 6. It can be seen that the filling ratio δ increased with the increase in ratio number $d_{p}h_{D}^{-1}$. This can primarily be attributed to two aspects. On the one hand, the increase in $d_{p}h_{D}^{-1}$ indicated that the particle size increased faster than the depth of die $h_{D}$. Due to being heavier and having a larger inertia, larger particles generally have better flowability than smaller ones. On the other hand, air escaped more easily and quickly in shallower dies than deeper ones, which made the particles less sensitive to air. Therefore, the volume flow rate of particles also rose with the increase in $d_{p}h_{D}^{-1}$, as shown in Figure 7, which effectively explains the above law in Figure 6.

**Figure 8:**
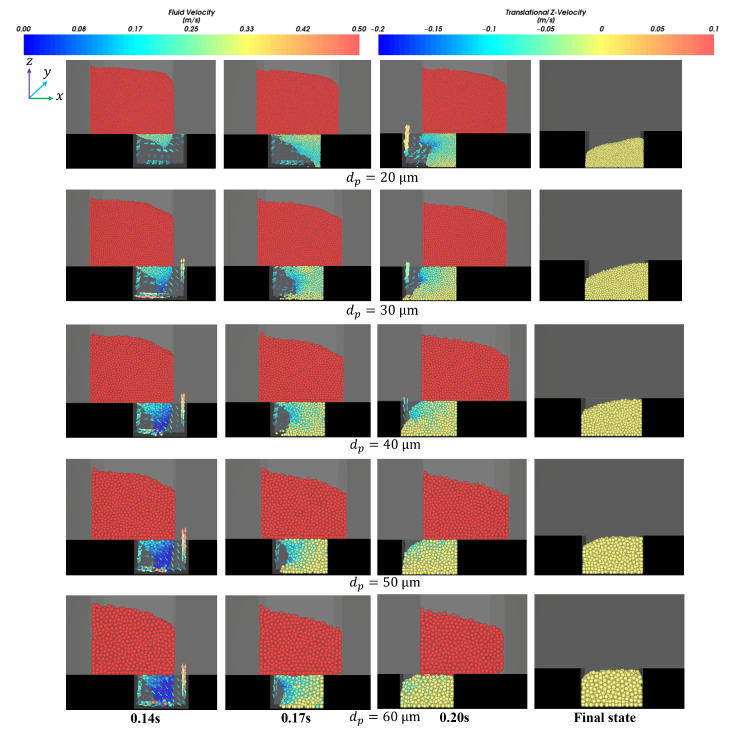
The distribution of particle velocity and air velocity under the conditions that the shoe moving speed is 0.1 m/s, the solid density is 7360 kg/m^3^, and the particle size varies from 20 μm to 60 μm.

It can also be seen from Figure 6 and Figure 7 that the filling ratio and volume flow rate changed slowly when the shoe speed number $v\rho_{g}L_{D}\mu_{g}^{-1}$ was no more than 68.5 (that means the shoe speed v≤0.125 m/s). In this case, the die was almost filled fully when the ratio number $d_{p}h_{D}^{-1}$ was over 0.007. The above law can be described and explained vividly in Figure 8. It can be seen that the influence of the air inside the cavity on filling was increasingly insignificant with the increase in particle size. In addition, fine particles induce larger air vortices, thereby enhancing drag effects on the particles. Cavities containing finer particles generate stronger air vortices, leading to a reduced filling capacity within equivalent time intervals, as shown in Figure 8.

### 3.2. The Shoe Speed Number

**Figure 9:**
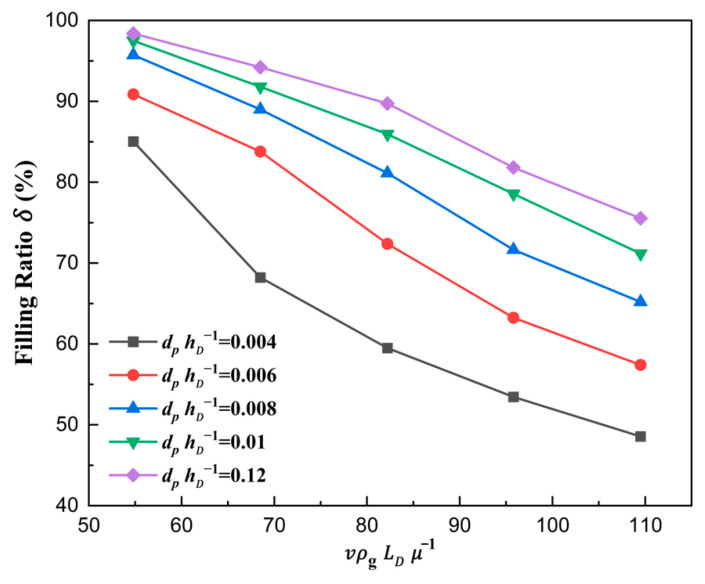
The filling ratio δ in different shoe speed numbers for $\rho_{p}\rho_{g}^{-1}=6008$.

The curve is plotted in Figure 9, where the shoe speed number $v\rho_{g}L_{D}\mu_{g}^{-1}$ affecting the filling ratio δ is analyzed quantitatively.

It can be seen that with the increase in $v\rho_{g}L_{D}\mu_{g}^{-1}$, the filling ratio δ decreased gradually. On the one hand, the effective time of die filling was reduced when the shoe speed number increased. On the other hand, the airflow, which hindered the particles from falling to some extent, could not escape before the shoe completely covered the die inlet due to the faster shoe speed. Therefore, the filling ratio δ decreased when the shoe moving speed increased.

**Figure 10:**
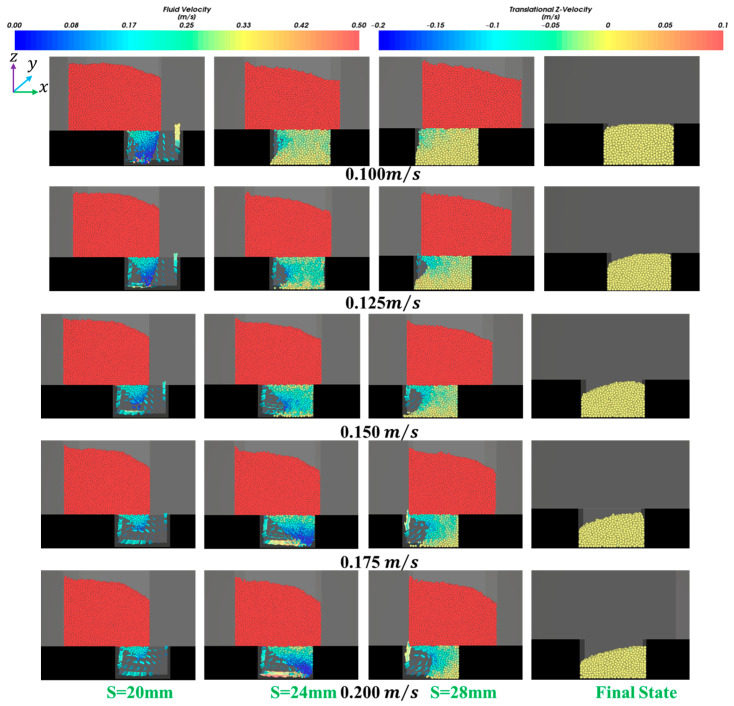
The distribution of particle velocity and air velocity under the conditions that the particle size is 40 μm, the solid density is 7360 kg/m^3^, the shoe speed varies from 0.100 to 0.125 m/s.

In Figure 10, a similar phenomenon to that in Figure 9 is observed. A higher air velocity was found when the shoe speed was higher at the same displacement. Therefore, the filling ratio δ decreased.

### 3.3. The Solid Density Number

**Figure 11:**
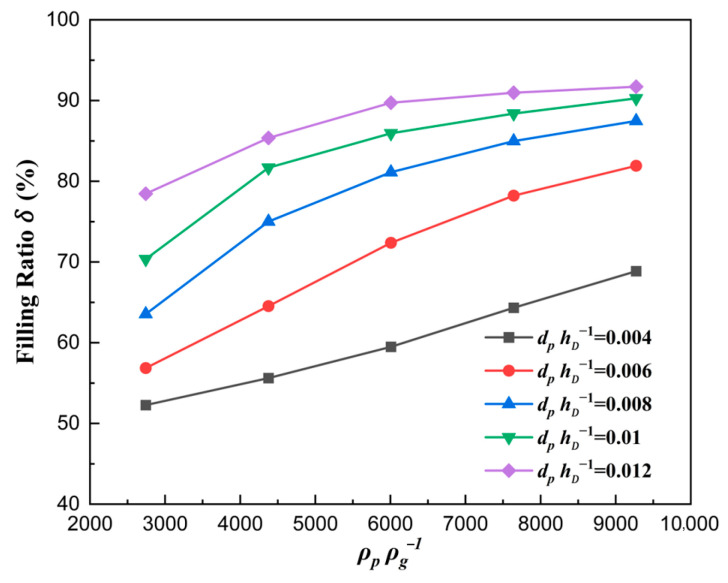
The filling ratio δ with different solid density numbers for $v\rho_{g}L_{D}\mu_{g}^{-1}=82.2$.

The curve is plotted in Figure 11, where the solid density number $\rho_{p}\rho_{g}^{-1}$ affecting the filling ratio δ is analyzed quantitatively. It can be seen that the filling ratio δ increased with the increase in solid density number $\rho_{p}\rho_{g}^{-1}$. The reason is that the particle’s gravity, as well as the acceleration and velocity in the falling direction, all increased, which would cause the number of particles filled in the die to increase at the same time.

**Figure 12:**
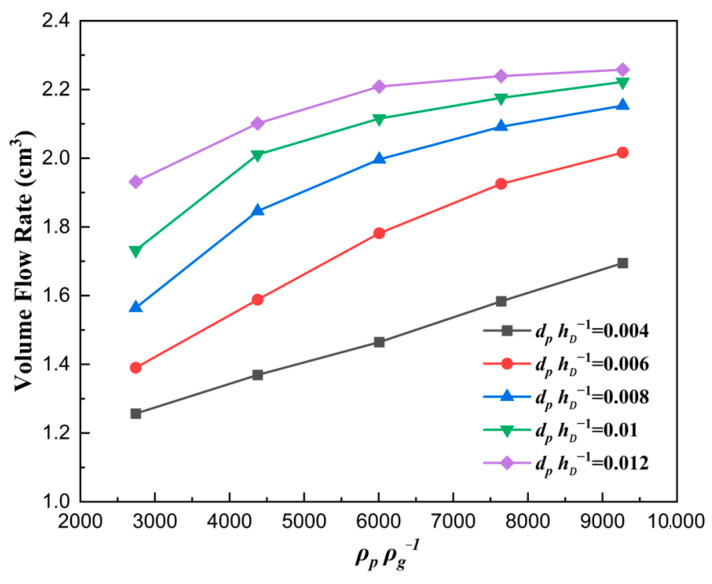
The volume flow rate of particles filled with different solid density numbers for $v\rho_{g}L_{D}\mu_{g}^{-1}=82.2$.

The curve describing the correlation between the volume flow rate and the solid density number $\rho_{p}\rho_{g}^{-1}$ is plotted in Figure 12. The volume flow rate of particles increased with the increase in solid density number, which verified the above explanation in Figure 11. A deeper analysis reveals that heavier particles are less sensitive to air, resulting in weaker hindering effects on heavier particles compared to lighter ones.

### 3.4. The Force Number

To explore the mechanism of linear die filling under the action of airflow inside the die, the forces on the particles were analyzed quantitatively, mainly including gravity and drag force. The force number $G_{p}F_{Drag}^{-1}$ was selected to explore the mechanism of die filling. The collisions among falling particles were neglected. Therefore, in the downward direction, that is, the negative direction of the Z axis, the force equation of particles obtained from Newton’s second law is as follows. Evidently, the acceleration $a_{z}$ determines the filling ratio and effects.

(20) $$m_{p}a_{z}=G_{p}-F_{Drag}$$

where $m_{p}$ and $a_{z}$ represent the single particle mass and the particle acceleration in the falling direction, respectively.

**Figure 13:**
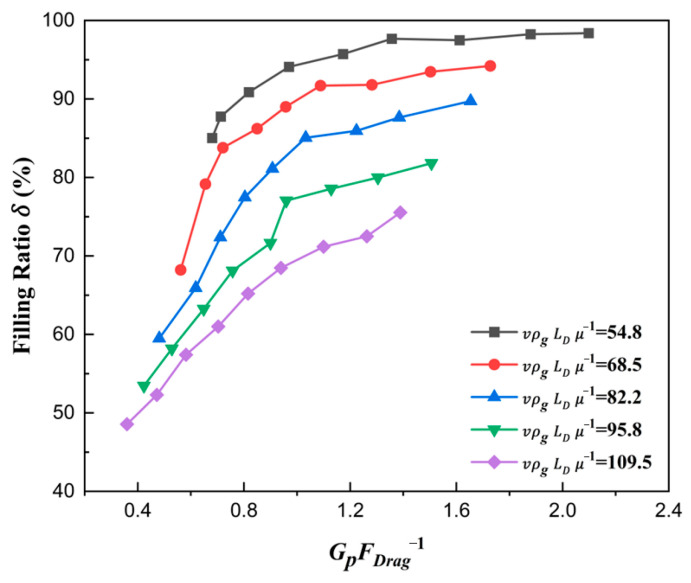
The filling ratio δ with different force numbers for $\rho_{p}\rho_{g}^{-1}=6008$.

The curve showing the relationship between the filling ratio *δ* and force number $G_{p}F_{Drag}^{-1}$ is plotted in Figure 13. It can be seen that the filling ratio δ increased with the increase in force number $G_{p}F_{Drag}^{-1}$. This can be explained by Equation (20). With the increase in $G_{p}F_{Drag}^{-1}$, the acceleration of particles in the falling direction also rose, which made the filling effects grow. And then, the number of particles falling in the die increased at the same time, and naturally, the filling ratio *δ* rose.

**Figure 14:**
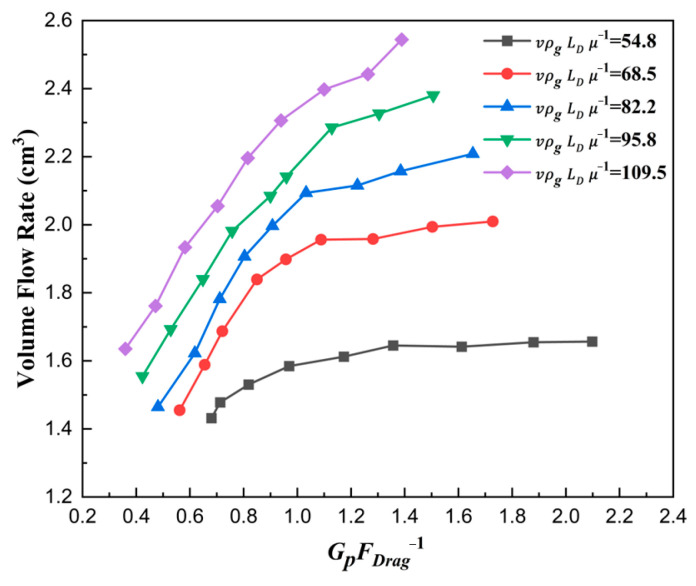
The volume flow rate with different force numbers for $\rho_{p}\rho_{g}^{-1}=6008$.

The volume flow rate of particles filled in the die is also described with the force number $G_{p}F_{Drag}^{-1}$ in Figure 14. With the increase in force number $G_{p}F_{Drag}^{-1}$, the volume flow rate rose apparently, which naturally explained the law in Figure 13. Therefore, only when the force number increases can the filling ratio increase.

**Figure 15:**
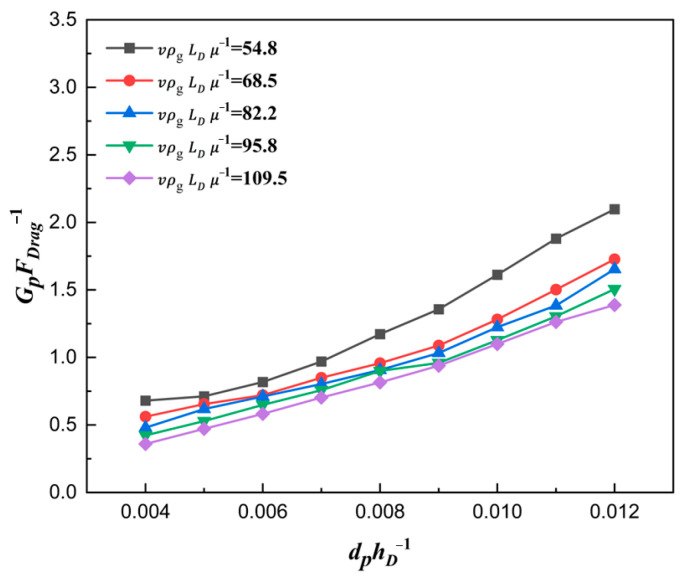
Force numbers for different ratios of particle size to die depth for $\rho_{p}\rho_{g}^{-1}=6008$.

**Figure 16:**
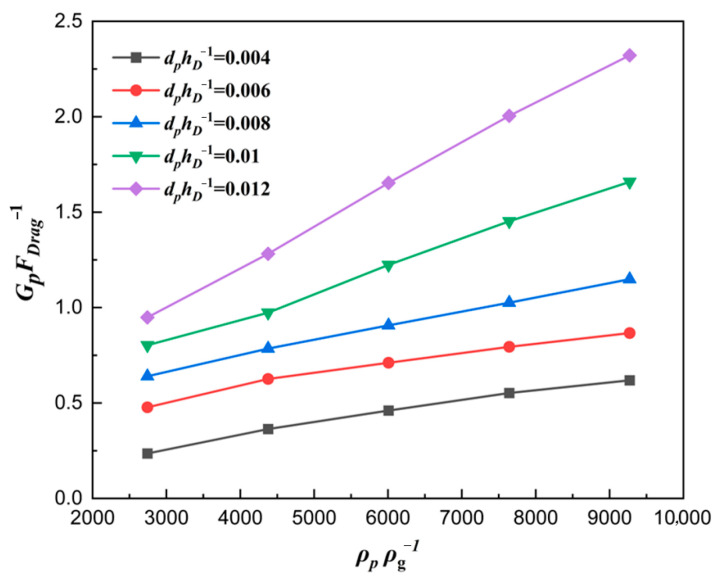
Force numbers for different material density numbers for $\rho_{p}\rho_{g}^{-1}=6008$.

The curve explaining the reason why a larger ratio number $d_{p}h_{D}^{-1}$ led to a larger filling ratio δ is plotted in Figure 15. As expected, with the ratio number $d_{p}h_{D}^{-1}$ increasing, the force number $G_{p}F_{Drag}^{-1}$ also increased, which was the essence of the filling ratio δ rising. Figure 16 shows the correlation between the force number $G_{p}F_{Drag}^{-1}$ and the solid density number $d_{p}h_{D}^{-1}$. With the increase in solid density number $\rho_{p}\rho_{g}^{-1}$, the force number $G_{p}F_{Drag}^{-1}$ increased considerably, which was the essence of the filling ratio δ rising affected by solid density. The main reason was that the gravity increased faster than the drag force, which caused a larger acceleration $a_{z}$.

Therefore, the filling ratio δ increased with the increase in ratio number $d_{p}h_{D}^{-1}$ and solid density number $\rho_{p}\rho_{g}^{-1}$. The fundamental reason lay in the relative proportion of the drag force to the gravitational force on the particles. The lower drag force relative to the gravitational force led to a larger filling ratio δ. Furthermore, with the increase in shoe speed, the filling ratio decreased due to the effective filling time shortening and the stronger drag force in Figure 9 and Figure 14.

### 3.5. Geometric Parameters

**Figure 17:**
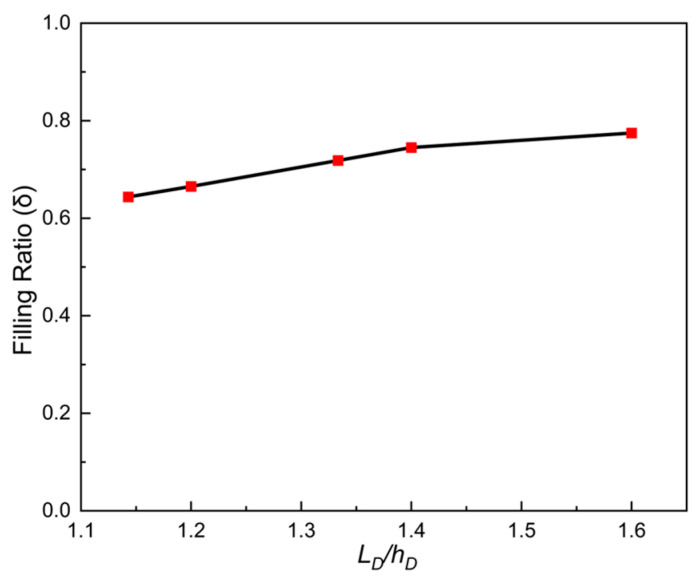
The filling ratio δ for different width-to-depth ratios of the die.

The macroscopic phenomenon of filling ratios among different geometric sizes of dies was difficult to distinguish. Therefore, quantitative analysis was conducted where the ratio $L_{D}/h_{D}$ of the opening size $L_{D}$ to the die depth $h_{D}$ was adopted to be variable, and the filling ratio δ was selected as an indicator. The curve showing the relationship between the ratio $L_{D}/h_{D}$ and filling ratio δ is plotted in Figure 17. It can be seen that δ increased with the increase in the ratio $L_{D}/h_{D}$. A larger opening size of the die contributed to the air inside the die escaping. The deeper die prolonged the time required for the air to be discharged from the die, which may have prevented the air from escaping completely before the opening was covered by the shoe. The air trapped in the die can block particles falling into the die. Therefore, the larger value of $L_{D}/h_{D}$ made the air escape faster. Naturally, the filling ratio would be larger with the increase in the ratio $L_{D}/h_{D}$.

### 3.6. Modeling of Linear Die Filling

**Figure 18:**
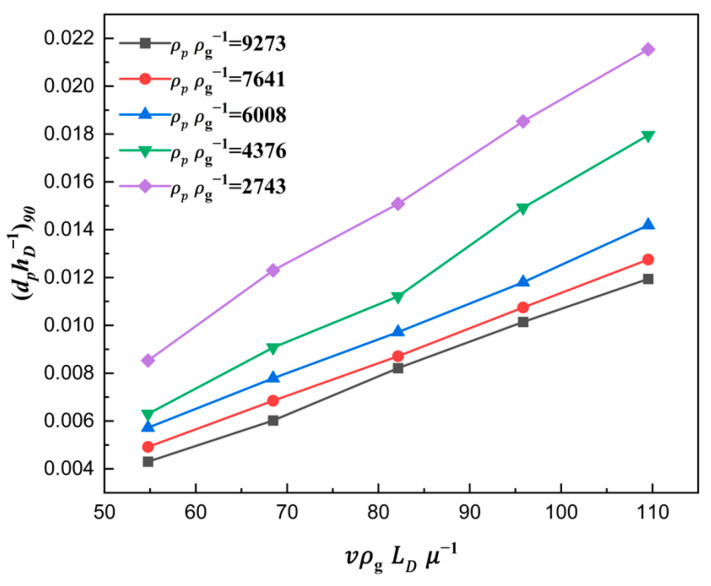
The critical value ${\left(d_{p}h_{D}^{-1}\right)}_{90}$ for different shoe speed numbers.

From the above analysis, we can see that the filling ratio δ rises slowly or even remains stable at the 90% percentage. Combining Equation (19), the ratio number $d_{p}h_{D}^{-1}$ was selected as the indicator of die filling when the filling ratio was 90%, which was treated as a sign of full filling, and then the corresponding critical value ${\left(d_{p}h_{D}^{-1}\right)}_{90}$ was suggested. A curve taking the critical value ${\left(d_{p}h_{D}^{-1}\right)}_{90}$ as the dependent variable and the solid density number $\rho_{p}\rho_{g}^{-1}$ and shoe speed number $v\rho_{g}L_{D}\mu_{g}^{-1}$ as independent variables is plotted in Figure 18. It can be seen that the critical value ${\left(d_{p}h_{D}^{-1}\right)}_{90}$ linearly increased with the increase in shoe speed number $v\rho_{g}L_{D}\mu_{g}^{-1}$ and decreased with the increase in solid density number $\rho_{p}\rho_{g}^{-1}$. In addition, the interaction between the shoe speed number $v\rho_{g}L_{D}\mu_{g}^{-1}$ and the solid density number $\rho_{p}\rho_{g}^{-1}$ was not obvious. Therefore, the semi-empirical modeling of the critical value ${\left(d_{p}h_{D}^{-1}\right)}_{90}$ was developed by the method of multiple linear fitting as follows:

(21) $${\left(d_{p}h_{D}^{-1}\right)}_{90}=0.00256+1.77202\times 10^{-4}\cdot \left(v\rho_{g}L_{D}\mu^{-1}\right)-1.05607\times 10^{-6}\cdot \left(\rho_{p}\rho_{g}^{-1}\right)$$

**Figure 19:**
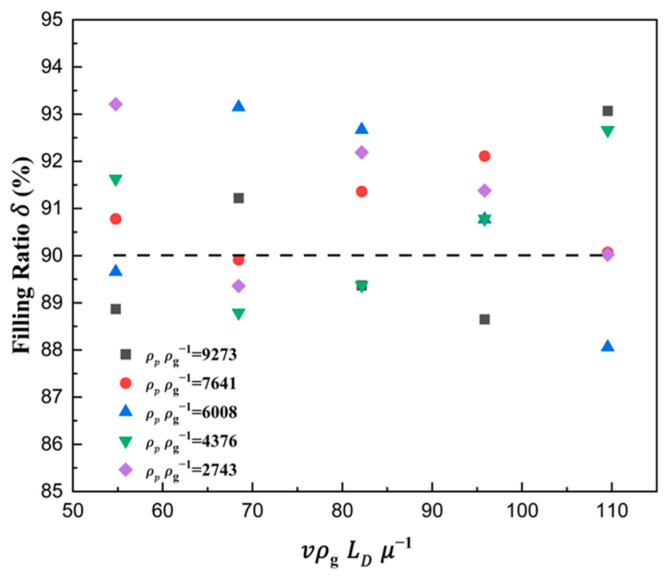
The predicted theoretical value and actual value in filling ratio δ, where the dotted line represent the 90% theoretical filling ratio.

The coefficient of determination of the model was greater than 0.95, and the verification of the model was conducted, as shown in Figure 19. It can be seen that the maximum error between the model and simulation results was no more than 3%. Therefore, the semi-empirical modeling of the critical value ${\left(d_{p}h_{D}^{-1}\right)}_{90}$ can be better used to predict and design the correlations among the particle properties, geometric parameters of the die, and the filling process.

In terms of particulate properties, lighter and finer particles exhibit heightened sensitivity to air flowing, resulting in compromised filling efficiency. From a process perspective, elevated shoe speeds impede sufficient air evacuation and shorten filling durations, collectively suppressing filling performance. Structurally, cavities with higher aspect ratios facilitate air escape, thereby enhancing filling efficacy. A semi-empirical model for complete filling was derived through dimensional analysis, offering practical guidance for achieving full compaction in powder-forming processes. However, this study focuses on regular mold geometries. For complex configurations, such as rhombic, stepped, or gear-shaped cavities, wall effects frequently lead to incomplete filling near boundaries. Developing robust methodologies to evaluate filling efficiency in such intricate molds remains a critical research frontier.

## 4. Conclusions

In this research, a critical value and a filling model were introduced to describe and predict the linear die filling results based on dimensional analysis derived from material properties, process parameters, and die geometrics. The details are as follows:

(1) The coarse-grain DEM-CFD method was adopted, and the scale-up factor was verified. The static and dynamic repose angles in experiments and DEM simulations were used to determine the contact parameters. The scale-up factor was also determined by DEM simulations in die filling.
(2) Based on dimensionless derivation and analysis, the ratio number of size $d_{p}h_{D}^{-1}$, the solid density number $\rho_{p}\rho_{g}^{-1}$, the shoe speed number $v\rho_{g}L_{D}\mu_{g}^{-1}$, and force number $G_{p}F_{Drag}^{-1}$ were derived. The filling ratio increased with the increase in $d_{p}h_{D}^{-1}$ and $\rho_{p}\rho_{g}^{-1}$ and decreased with the increase in $v\rho_{g}L_{D}\mu_{g}^{-1}$. The force number $G_{p}F_{Drag}^{-1}$ was found to be a key point determining the filling ratio. Due to the falling of the particles being hindered by the airflow, smaller proportions of drag forces relative to gravity result in larger filling ratios, which is the essence of filling.
(3) A filling ratio of ninety percent was selected as a sign of full filling, and then, the corresponding critical value ${\left(d_{p}h_{D}^{-1}\right)}_{90}$ was obtained. A semi-empirical modeling of linear die filling, taking the critical value ${\left(d_{p}h_{D}^{-1}\right)}_{90}$ as the dependent variable and solid density number $\rho_{p}\rho_{g}^{-1}$ and shoe speed number $v\rho_{g}L_{D}\mu_{g}^{-1}$ as independent variables, was developed. Hence, this model can be used to design the smallest shoe speed to achieve complete die filling, optimize the parameters of die structures, and support process development.

## Figures and Tables

**Table 1:** The simulation parameters.

The Solid		Symbol	Value
	Normal force model	$F_{n,i}$	Hertzian spring–dashpot model
	Tangential force model	$F_{t,i}$	Mindlin–Deresiewicz model
	Particle size (μm)	$d_{p}$	20~60
	Solid density (kg/m^3^)	$\rho_{s}$	3360~11,360
	Young’s modulus	E	$1\times 10^{8}$
	Poisson’s ratio	v	0.3
	Friction coefficient	μ	0.67
	Rolling resistance	r	0.218
	Restitution coefficients	ε	0.5
	CGM scale factor	k	1, 4, 6, 8, 10
The air			
	Density (kg/m^3^)	$\rho_{g}$	1.225
	Viscosity (Pa∙s)	$\mu_{g}$	$1.79\times 10^{-5}$
Process			
	Shoe speed (m/s)	v	0.100~0.200

**Table 2:** The filling ratio with different scale factors.

Scale-Up Factor	k=1	k=4	k=6	k=8	k=10
Filling ratio (%)	50.87	50.68	50.96	50.42	50.70

## Data Availability

The original contributions presented in this study are included in the article. Further inquiries can be directed to the corresponding author.
